# Face Gender Influences the Looking Preference for Smiling Expressions in 3.5-Month-Old Human Infants

**DOI:** 10.1371/journal.pone.0129812

**Published:** 2015-06-11

**Authors:** Laurie Bayet, Paul C. Quinn, James W. Tanaka, Kang Lee, Édouard Gentaz, Olivier Pascalis

**Affiliations:** 1 LPNC, University of Grenoble-Alps, Grenoble, France; 2 LPNC, CNRS, Grenoble, France; 3 Department of Psychological and Brain Sciences, University of Delaware, Newark, Delaware, United States of America; 4 Department of Psychology, University of Victoria, Victoria, British Columbia, Canada; 5 Dr. Eric Jackman Institute of Child Study, University of Toronto, Toronto, Ontario, Canada; 6 Faculty of Psychology and Educational Sciences, University of Geneva, Geneva, Switzerland; Brock University, CANADA

## Abstract

Young infants are typically thought to prefer looking at smiling expressions. Although some accounts suggest that the preference is automatic and universal, we hypothesized that it is not rigid and may be influenced by other face dimensions, most notably the face’s gender. Infants are sensitive to the gender of faces; for example, 3-month-olds raised by female caregivers typically prefer female over male faces. We presented neutral versus smiling pairs of faces from the same female or male individuals to 3.5-month-old infants (*n* = 25), controlling for low-level cues. Infants looked longer to the smiling face when faces were female but longer to the neutral face when faces were male, i.e., there was an effect of face gender on the looking preference for smiling. The results indicate that a preference for smiling in 3.5-month-olds is limited to female faces, possibly reflective of differential experience with male and female faces.

## Introduction

Faces are complex hierarchical stimuli displaying much information at once. The original Bruce and Young model [1] postulates that variant (expression, gaze, speech movements) and invariant (identity, race, gender) dimensions are separated during the structural encoding stage of face perception; an early-stage pictorial code (or snapshot) converts into a set of expression-independent representations for each view, simultaneously resulting in the extraction of variant features that are independently streamlined to process speech movements and facial expressions. Invariant dimensions then arise at the semantic level from an integration of expression-independent representations, view-centered representations of particular features, and other inputs. A recent adaptation of this model proposed by Haxby, Hoffman, and Gobbini [2] suggests a more symmetrical division between variant and invariant aspects of faces, with possible interactions between both streams at the perceptual level. Indeed, in human adults there is evidence that variant and invariant face dimensions interact relatively early, even in subcortical structures [3]. For example, facial expressions can influence face recognition in adults, with a smile acting like a cue to familiarity [4].

One example of variant facial dimension perception in infancy is the preference for smiling faces that is sometimes reported in infants younger than 5 months; newborns look longer at smiling over fearful faces [5] and 4-month-olds prefer smiles to other facial expressions [6]. This early preference for smiling faces is not stable during development (older infants look longer to fearful faces instead [7]), and its cause remains unclear as sensitivity to other types of emotional expressions (e.g., fear) doesn’t emerge until later around 5–7 months of age [8]. Young infants may prefer the salience of teeth [9], perceive smiles as positive because they mirror them via affect matching [10–12], or are equipped with a basic universal module for emotion recognition [13,14]. It is also possible that infants prefer the expression most familiar to them, given that caregivers tend to display faces depicting positive affect [15]. Similarly, infants may come to prefer smiling faces because they tend to signal the onset of positive interactions with caregivers which are inherently rewarding (i.e., classical conditioning). Strikingly, it has been reported that 3-month-old infants recognize a face better when the face is smiling dynamically during familiarization [16,17], suggesting a possible influence of expression on identity perception.

We posited that the perception of smiling by young infants is integrated into the face perception system, so that it may be influenced by other facial dimensions, and particularly by the dimension of gender. Face gender is a salient dimension in infancy, as gender differences in nonverbal communication and caretaking [18,19] cause systematic differences in the relative familiarity of infants with male and female faces. Most infants are indeed primarily raised by a female caregiver and experience fewer male than female faces during their first year [20,21]. Infants may thus react differently to male and female smiles. For example, if infants prefer smiling faces because caregivers tend to display faces depicting positive affect [15], and if most infants are raised by female caregivers [20,21], it follows that infants may prefer female smiles more than male smiles.

**Fig 1:**
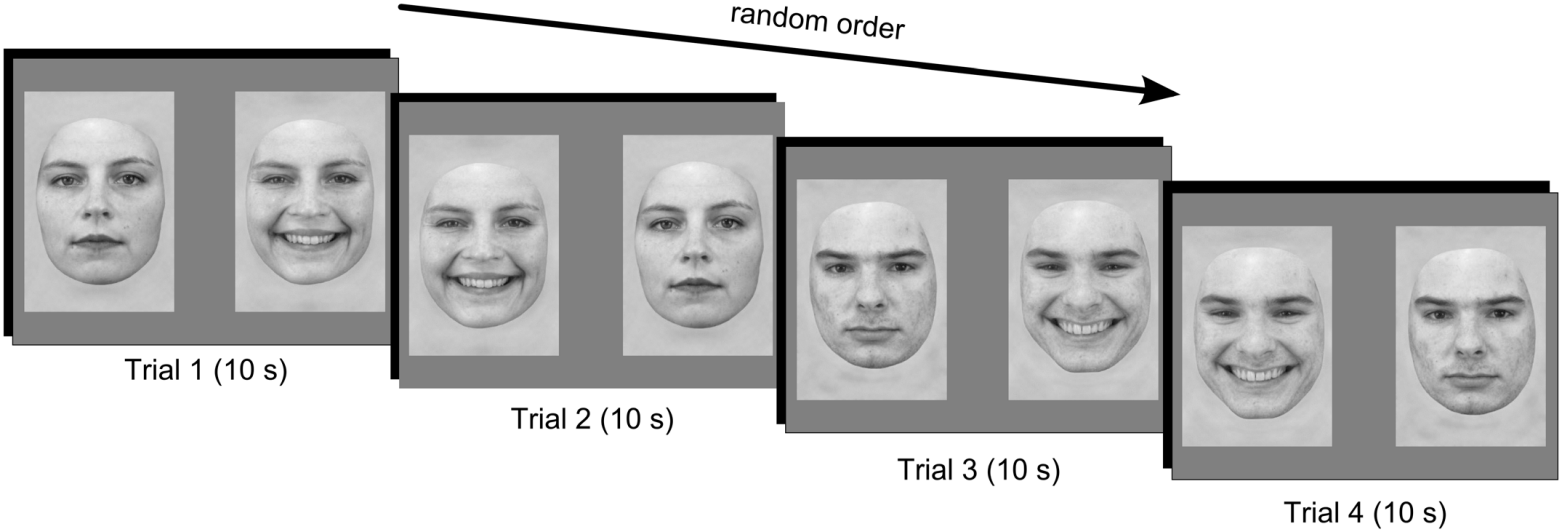
Example session. Each infant saw all four trials, featuring stimuli from one of two stimulus sets.

Our study aimed to test the effect of face gender on the looking preference of infants for smiling expressions. We presented male and female smiling faces paired with neutral faces of the same individual to 3.5-month-old infants (Fig 1, *n* = 25), an age at which a preference for smiling has been reported [6]. Low-level properties of the faces were equated, two different stimulus sets were used (S1 Table), and looking preferences were measured. Some accounts of the preference for smiling faces in young infants based on salience [9], mimicking [10–12], or a module for emotion recognition [13,14] would predict that face gender is irrelevant to eliciting preferential responding to smiling faces, and that infants should prefer both male and female smiles. However, given that the parental distribution of caregiving has been found to modulate the reaction of 14-month-olds to emotional expressions displayed by their mother and father [22], and given the increased familiarity of infants with female faces, it is also possible that the preference for smiling facial expressions would be greater, or at least more robust, in female faces than in male faces.

## Methods

### Participants

Twenty-five 3.5-month-old infants (13 girls, mean age 115.4 ± 5.6 days, range 101–122 days) were included in the study. All caregivers gave informed written consent before testing, and all infants were born full term (39.1 ± 1.2 weeks of amenorrhea). Ten infants were excluded due to fussiness (*n* = 3), technical failure (*n* = 2), or experimental error (*n* = 5). Thirty-seven infants were excluded due to side-bias, i.e. they looked in one direction more than 95% of the time in one or more of the four trials. Although the attrition rate was high, the definition and handling of side biases was decided a priori, in accordance with common methods in infant research [28], and motivated by the need to run within-participant comparisons between male and female pairings. Means for each condition were substantially unchanged if data points from those excluded participants that had no side bias for the male (*n* = 10) or female pairing (*n* = 8) were included. All caregivers reported a percent of female caretaking of at least 50% (mean 69 ± 2%), meaning that no infant in our sample was primarily raised by a male caregiver, and providing results that are consistent with those previously reported [20,21].

### Stimuli

Two sets of stimuli were used that had different face identities (sets A and B, S1 Table). Stimulus set was counterbalanced across infants (12 of the 25 3.5-month olds viewed set A). Face stimuli were selected from the Karolinska Directed Emotional Face database [29,30] under their smiling and neutral frontal view versions. They were gray scaled; external features were cropped. Luminance, contrast, spatial frequencies, and placement of the eyes were matched using SHINE [31] and Psychomorph [32] for each set. Faces subtended a visual angle of about 9 degrees (vertically) by 7 degrees (horizontally). Physical and emotional properties of the stimuli are summarized in S1 Table.

### Procedure

We presented male or female smiling faces paired with neutral faces of the same individual to 25 3.5-month-old infants (Fig 1) using E-Prime 2.0 [33]. The infants sat on their caregiver’s lap about 60 cm from a screen. Each infant saw 4 trials showing 1 female and 1 male pair of faces. There were 2 trials for each pair, with left-right side of presentation reversed. The 4 trials were randomly ordered and lasted 10 s from first look. The infant’s gaze was redirected to the center of the screen between each trial. The experiment was approved by the local ethics committee (“Comité d’éthique des centre d’investigation clinique de l’inter-région Rhône-Alpes-Auvergne”, Institutional Review Board).

### Data acquisition, pre-processing, and analysis

Infant looking was recorded by a camera and coded off line with 40 ms precision (25 frames per second). A sub-sample of the videos was coded by a second observer with 0.98 agreement (Pearson’s *r*, 24% of the videos). Analyses were run in Matlab R2009b using the Statistics toolbox. Looking preferences towards each stimulus were derived from looking times (Percentage of Total Looking Time, PTLT). For example, the looking preference (PTLT) for the smiling female face for each infant was created by averaging the percentage of looking time to the smiling female face (versus the neutral female face) in the two trials featuring female stimuli. PTLTs to male (3 infants) or female faces (1 infant) that were further than 2 standard deviations away from the corresponding group mean were considered outliers and excluded (8% of trials). The handling of outliers was decided a priori and in accordance with common methods in infant research [34,35]. There was no significant difference in the mean total looking times during male and female trials (paired Student’s *t*-tests, *t*[20] = 1.16, *p* = 0.261).

## Results

### An effect of face gender on the looking preference for smiling

A preliminary ANOVA revealed no effect of the two between-participant factors of stimulus set (*F*[1,17] = 0.06, *p* = 0.809) or participant gender (*F*[1,17] = 0.79, *p* = 0.386) or their interaction (*F*[1,17] = 1.37, *p* = 0.258) on the looking preference (PTLT) for the smiling expression. Three similar, preliminary ANOVAs on the looking preferences (PTLT) for female and male smiles and on the difference between them also revealed no effect of the between-subject factors of stimulus set, participant gender, or their interaction (all *p*s > 0.05). Consequently, data from all participants were pooled together.

**Fig 2:**
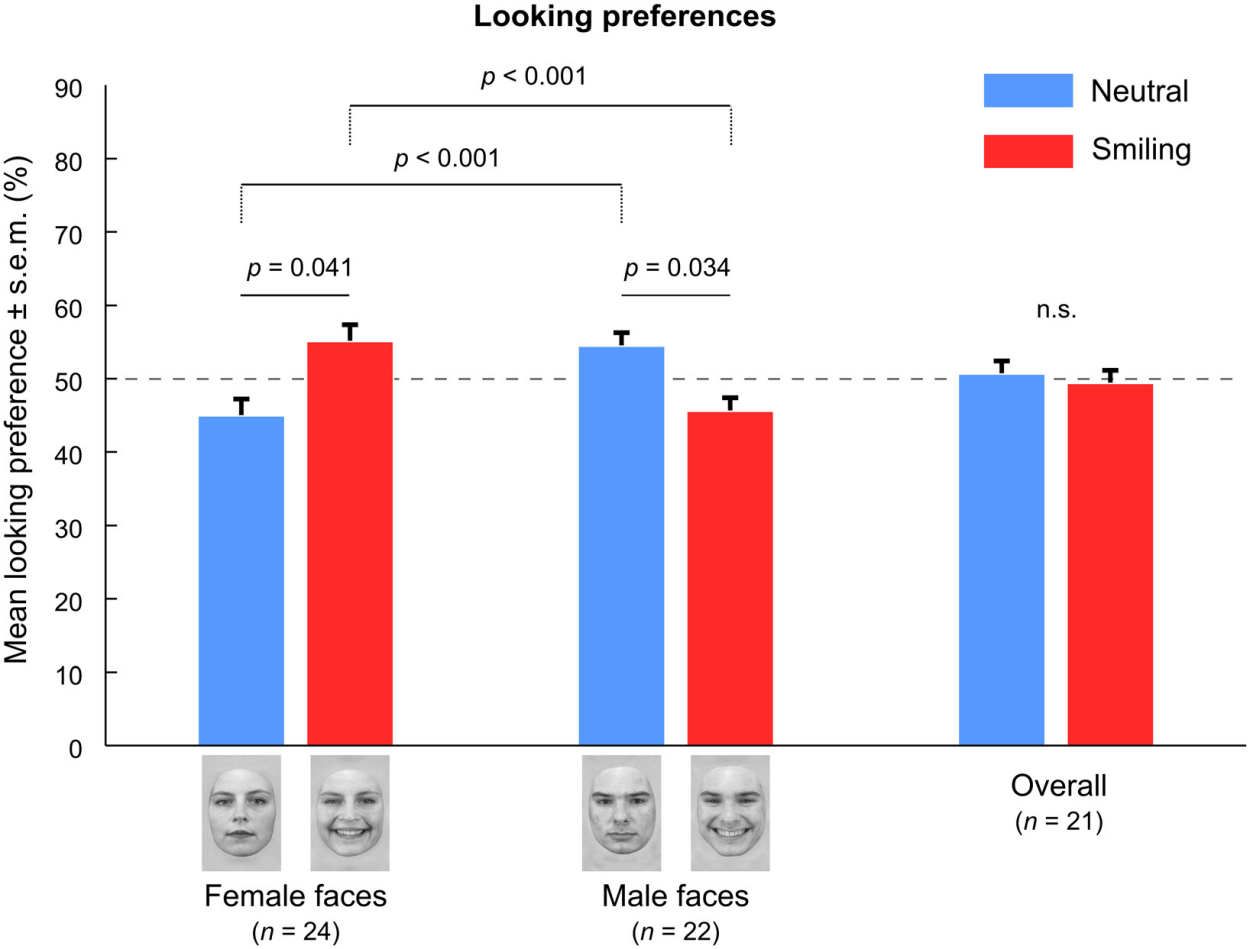
Face gender influences the looking preference for smile. A looking preference of 50% represents chance level. Overall, infants preferred looking to the smiling face in female pairings, and to the neutral face in male pairings (as measured by Percentages of Total Looking Time, PTLT). There was no overall preference when pooling female and male trials together. Paired Student *t*-tests, α = 0.05, uncorrected.

**Fig 3:**
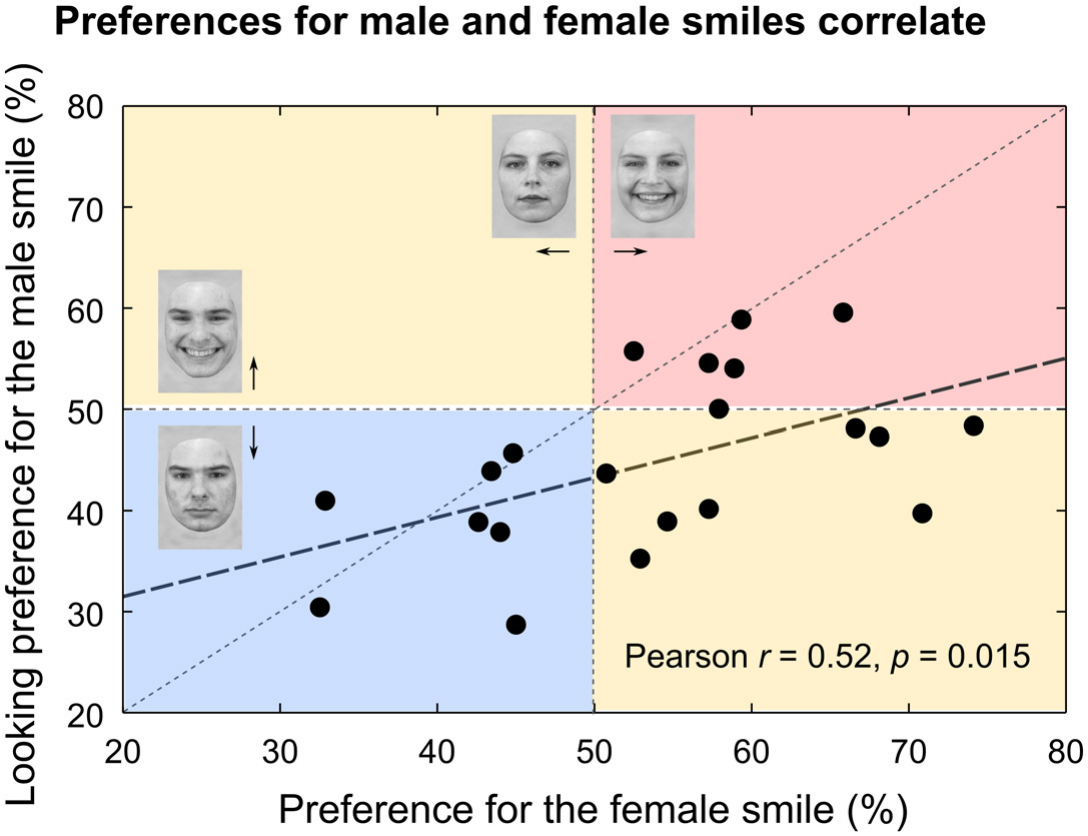
Individual looking preferences for male and female smiles correlate. Most infants showed a stronger preference for the smiling face on female face trials (i.e., smiling vs. neutral female face) than on male face trials (i.e., smiling vs. neutral male face), as is indicated by the position of the regression line below the identity line.

Infants did not look longer at the smiling than at the neutral faces (*t*[20] = -0.33, *p* = 0.746, paired Student’s *t*-test). A repeated-measure ANOVA on the looking preference for the smiling expression further revealed a significant effect of face gender (*F*[1,20] = 16.68, *p* < 0.001). Infants looked longer to the smiling female face versus neutral female face (*t*[23] = 2.16, *p* = 0.041, Cohen’s *d* = 0.44, Fig 2, Student’s *t*-test against chance level, uncorrected), but longer to the neutral male face versus smiling male face (*t*[21] = -2.27, *p* = 0.034, Cohen’s *d* = -0.48, Fig 2, Student’s *t*-test against chance level, uncorrected). They also looked longer at the smiling expression when the faces were female than when they were male, and conversely longer at the neutral expression when the faces were male than when the faces were female (both comparisons: *t*[20] = 4.08, *p* < 0.001, Cohen’s *d* = 0.89, Fig 2, paired Student’s *t*-test, uncorrected). Looking behavior was consistent across individuals; 17 out of 21 infants showed a stronger preference for the smiling face on female face trials (i.e., smiling vs. neutral female face) than on male face trials (i.e., smiling vs. neutral male face; Fig 3, 17 out of 21 data points are below the identity line). Seventeen out of 24 infants looked longer to the smiling versus neutral female faces, while 7 out of 22 infants looked longer to the smiling versus neutral male faces.

### A correlation of individual looking preferences for male and female smiles

The pattern of opposite preference for smiling in male and female faces suggests that 3.5-month-old infants process male and female smiles independently. However, individual looking preferences to male and female smiles correlated significantly (Pearson’s *r* = 0.53, *p* = 0.015, Fig 3). Regression analyses (one per factor) revealed no effect of stimulus set or participant gender in this relationship, either as a main effect or in interaction with looking preferences (all *p*s > 0.05). Infants at 3.5 months of age process male and female smiles using partly common mechanisms, but seem to consistently prefer the female smile more than the male smile.

## Discussion

Contrary to predictions from saliency [9], mimicking via affect matching [10–12], and universal emotion recognition accounts [13,14], face gender modulated the response of 3.5-month-olds to the smiling facial expression. The correlation of individual preferences for male and female smiles suggests partially common mechanisms in the processing of male and female smiles, which is in keeping with saliency, mimicking, or universal facial expression processing accounts; however, the female smile was systematically more preferred than the male smile, sharply contrasting with the predictions from those accounts. Moreover, at the group level, a preference for smiling was only found for female faces, evidencing the dependence of expression perception on broader face processing. While several studies have reported interactive effects of eye gaze and expression [23,24] or eye gaze and facial identity [25] in 3- to 4-month-olds at the electrophysiological level, this is the first study reporting an effect of face gender on behavioral responses to facial expression in this population.

### Experience shapes the response of infants to smiling faces

Younger infants have limited perceptual and social experience with male faces [20,21], which could lead to differential processing of male and female facial expressions in at least two ways. First, adult females may smile more than males when interacting with infants [18]; the looking preferences of infants for female smiles and male neutral expressions would thus represent a primitive form of stereotyping based on familiarity. Second, young infants primarily raised by a female could tend to see females, but not males, as potential caregivers; this could lead them to respond more to a female than to a male smile. In adults, a smile’s positive value depends on the relationship shared between the observer and the person smiling [26] and might stem from it being an affiliative cue [4]. Finally, Quinn et al. have argued that the social character of stimuli influences infant responding to particular characteristics of those stimuli [27]; infants may perceive male faces as less social than female faces.

### Conclusions

Infants at 3.5 months of age show different, but not independent, preferences for male and female smiles. They prefer looking to smiling (versus neutral) female faces and to neutral (versus smiling) male faces, although individual preferences for male and female smiles correlate. Thus, the preference for smiling by 3.5-month-old infants is neither universal nor automatic, but is already shaped by experience. Indeed, the data present an effect of face gender on smiling preference that possibly stems from the association of female faces with positive expressions and from the lack of perceptual and social experience infants have with male faces. The modulation of this effect by static versus dynamic smiles, its evolution during development, and its presentation in infants primarily raised by male caregivers as well as in newborns (in which a smiling preference may well be independent of experience) all remain to be tested in future research. We predict that infants raised primarily by a male caregiver would show a reverse pattern of preference, i.e., a preference for smiling versus neutral male faces and for neutral versus smiling female faces.

## Supporting Information

S1 Table Stimulus properties. **A.** Physical properties. Pixel values are approximate. Differences between male and female faces used here can be noted. Male faces enlarge more with smiling, and have smaller eyes but bigger teeth. Both male and female faces get wider with smaller eyes when smiling. **B.** Emotional properties from a validation study in adults [30]. All stimuli adequately conveyed the desired emotion. Differences between sets were greater than differences between male and female faces within each set. Hit rates, intensity, and arousal ratings are typical of neutral and smiling faces [30].
(XLSX)
